# Interleukin-2 Therapy of Autoimmunity in Diabetes (ITAD): a phase 2, multicentre, double-blind, randomized, placebo-controlled trial

**DOI:** 10.12688/wellcomeopenres.15697.1

**Published:** 2020-03-20

**Authors:** M. Loredana Marcovecchio, Linda S. Wicker, David B. Dunger, Susan J. Dutton, Sylwia Kopijasz, Claire Scudder, John A. Todd, Paul R. V. Johnson

**Affiliations:** 1 Department of Paediatrics, University of Cambridge, Cambridge, CB2 0QQ, UK; 2 JDRF/Wellcome Diabetes and Inflammation Laboratory, Nuffield Department of Medicine, Wellcome Centre for Human Genetics, NIHR Oxford Biomedical Research Centre, University of Oxford, Oxford, OX3 7BN, UK; 3 Wellcome Trust-MRC Institute of Metabolic Science, University of Cambridge, Cambridge, CB2 0QQ, UK; 4 Oxford Clinical Trials Research Unit, Centre for Statistics in Medicine, Nuffield Department of Orthopaedics, Rheumatology and Musculoskeletal Sciences, University of Oxford, Oxford, OX3 7LD, UK; 5 Islet Transplant Research Group, Nuffield Department of Surgical Sciences, Centre for Diabetes, Endocrinology and Metabolism (OCDEM), University of Oxford, Oxford, OX3 9DU, UK

**Keywords:** type 1 diabetes, children, adolescents, immunotherapy, Interleukin-2, aldesleukin, Beta cells, C-peptide, Treg

## Abstract

Type 1 diabetes is a common autoimmune disease due to destruction of pancreatic β cells, resulting in lifelong need for insulin. Evidence suggest that maintaining residual β-cell function can improve glucose control and reduce risk of hypoglycaemia and vascular complications.

Non-clinical, preclinical and some preliminary clinical data suggest that low-dose interleukin-2 (IL-2) therapy could block pancreatic β cells destruction by increasing the number of functional regulatory T cells (Tregs) that inhibit islet-specific autoreactive effector T cells (Teffs). However, there is lack of data on the effect of low-dose IL-2 in newly diagnosed children and adolescents with T1D as well as lack of specific data on its potential effect on β-cell function.

The ‘ **I**nterleukin-2 **T**herapy of **A**utoimmunity in **D**iabetes (ITAD)’ is a phase 2, multicentre, double-blind, randomised, placebo-controlled trial in children and adolescents (6-18 years; having detectable C-peptide) initiated within 6 weeks of T1D diagnosis. A total of 45 participants will be randomised in a 2:1 ratio to receive either ultra-low dose IL-2 (aldesleukin), at a dose of 0.2 x 10 ^6^ IU/m ^2^ twice-weekly, given subcutaneously, or placebo, for 6 months.

The primary objective is to assess the effects of ultra-low dose aldesleukin administration on endogenous β-cell function as measured by frequent home dried blood spot (DBS) fasting and post-prandial C-peptide in children and adolescents with newly diagnosed T1D. The secondary objectives are: 1) to assess the efficacy of regular dosing of aldesleukin in increasing Treg levels; 2) to confirm the clinical safety and tolerability of ultra-low dose aldesleukin; 3) to assess changes in the immune system indicating benefit or potential risk for future gains/loss in β-cell function and immune function; 4) to assess treatment effect on glycaemic control.

Trial registration: EudraCT 2017-002126-20 (06/02/2019)

## List of abbreviations

AE: adverse event

AR: adverse reaction

BMI: body mass index

BP: blood pressure

cGVHD: chronic graft-versus-host-disease

CRF: case report form

DBS: dried blood spot

GCP: good clinical practice

HbA1c: haemoglobin A1c

HR: heart rate

IL-2: interleukin-2

IL2RA: IL-2 receptor

IMP: investigational medical product

ITAD: Interleukin-2 Therapy of Autoimmunity in Diabetes

MHRA: Medicines and Healthcare products Regulatory Agency

MMTT: mixed meal tolerance test

NK: natural killer

OCTRU: Oxford Clinical Trials Research Unit

PBMC: peripheral blood mononuclear cell

RRAMP: Registration/Randomisation and Management of Product

SAE: serious adverse events

SAR: serious adverse reactions

SmPC: summary of product characteristics

SUSARs: Suspected Unexpected Serious Adverse Reactions

TBNK FACS: T-cell, B-cell and NK cell Fluorescence Activated Cell Sorting

Teff: T effector cells

Treg: T regulatory cells

T1D: type 1 diabetes

## Introduction

### Rationale for IL-2 treatment in type 1 diabetes

Type 1 diabetes (T1D) is a common autoimmune disease primarily mediated by T cells responses against pancreatic islet β-cell autoantigens, leading to the destruction of β cells and lack of insulin secretion ^1, 2^. Insulin remains the mainstay of treatment for T1D, although even the most advanced insulin delivery technologies do not fully replace the benefits of endogenous insulin-secreting β cells ^1^.

Most newly diagnosed patients still have sufficient insulin production to reduce risk of acute complications of T1D, such as hypoglycaemia, and long-term vascular complications ^3, 4^. If this residual endogenous insulin secretion could be preserved, by inhibiting the autoimmune attack and improving β-cell fragility to a hostile immune and hyperglycaemic environment, then many of these life-threatening complications could be potentially avoided and exogenous insulin requirements reduced ^4^.

The interleukin-2 (IL-2) pathway is a key genetically-validated pathway with potential therapeutic applications to T1D ^5, 6^. IL-2 is an essential molecule for immune homeostasis, necessary for the expansion and function of the CD4 ^+^ FOXP3 ^+^ T regulatory cells (Tregs) that sustain self-tolerance and prevent autoimmunity, including that induced by anti-β-cell T effector cells (Teff) ^7, 8^. Autoreactive Teffs are implicated in T1D pathogenesis, and susceptibility to T1D is due to less efficient regulation of Teffs by Tregs, and Teff anti-islet reactivity and activation ^9^. IL-2 binds to the heterotrimeric IL-2 receptor (IL2RA), which consists of CD25 (α chain) encoded by the *IL2RA* gene, CD122 (β) and CD132 (γ) ^10^. Genetic susceptibility at *IL2RA* is complex where genotype-to-phenotype studies indicate different effects of distinct disease-associated *IL2RA* alleles on different T cell subsets, including reduced sensitivity of Tregs to IL-2 ^10, 11^. The ability to respond to IL-2 differs between Treg and Teff cells, due to different expression levels of CD25 and the balance of their intracellular signalling molecules: Tregs have a ten-fold greater sensitivity to IL-2 compared to Teff cells, due to higher expression of the IL-2 receptor ^6^. This opens a therapeutic window for the use of ultra-low doses of IL-2 to enhance selectively the Treg response in patients with T1D or other autoimmune and inflammatory diseases ^12^. IL-2 therapy is currently being investigated in many diseases, including T1D ^13– 30^ and our own investigations have established the doses of IL-2 in T1D patients that stimulate Tregs but not Teffs ^23, 24^.

### Trials using low or ultra-low dose IL-2

Aldesleukin is a commercially available IL-2 produced by recombinant DNA technology, which differs from the natural cytokine by the absence of glycan residues, presence of a serine instead of a cysteine at position 125 and absence of the N-terminal alanine ^31^. Aldesleukin at low dose, in the order of 1.0 × 10 ^6^ IU per day ^15, 16, 29, 30^, or ultra-low dose, less than 1.0 × 10 ^6^ IU per week, in adults and children ^17, 18, 23, 24^, induces Tregs in a dose-dependent manner with no drug-related adverse events except for a small temporary non-itchy rash at the site of injection ^22– 24^.

Aldesleukin has been used for over 20 years, mainly in patients with cancer or HIV at very high doses, tens of millions of units daily, often delivered intravenously ^32, 33^. More recently, following promising results from preclinical studies, aldesleukin at low or ultra-low doses has been successfully and safely used in trials for the treatment of chronic graft-versus-host-disease (cGVHD) ^26, 27, 30^ and hepatitis C virus-induced vasculitis ^16^.

The first study in cGVHD used subcutaneous doses of aldesleukin of 0.3 × 10 ^6^, 1.0 × 10 ^6^, 3.0 × 10 ^6^ IU/m ^2^ daily for 8 weeks ^30^. In all patients, treatment led to an increased numbers of CD4 Tregs without an increase in Teff cells and patients showed persistent clinical responses with extended therapy ^30^. In a study including patients with hepatitis C virus-induced vasculitis, aldesleukin was administered subcutaneously at an initial dose of 1.5 × 10 ^6^ IU/day for 5 days, and then three additional doses at 3.0 × 10 ^6^ IU/day for 5 days at 3-weekly intervals ^16^. In these patients, low dose IL-2 was safe, with the highest tolerated dose in the cGvHD study of 1.0 × 10 ^6^ IU/m ^2^ daily. Additional studies have been performed with low dose aldesleukin and they have also included patients with autoimmune conditions, such as systemic lupus erythemathosus ^19, 20, 34^ and alopecia areata ^21^. Overall these studies have highlighted effective dosing of 0.3 - 1.0 × 10 ^6^ IU of aldesleukin subcutaneously, which was administered at a variable frequency across studies (daily, cycles of three times per week, or 5-day induction and 3-weekly maintenance). Therapy was well tolerated, with only minor reactions at the injection sites, and was associated with Treg expansion in a dose-dependent manner in most patients without T-cell expansion. Clinical outcomes improved in many patients.

In a phase 2 paediatric trial, ultra-low dose aldesleukin (1.0 to 2.0 × 10 ^5^ IU/m ^2^ three times per week) was administered in 16 patients after allogeneic hematopoietic stem cell transplantation to evaluate the effect on Treg immune reconstitution and thus prevent moderate to severe GVHD without increasing the risk of viral infections or relapse ^18^. Treatment for 6 and up to 12 weeks was associated with increased Tregs, from a mean (range) of 4.8% (0–11.0%) pre-IL-2 to 11.1% (1.2–31.1%) post-therapy. This on average increase in Tregs of over 100% was well tolerated without increasing the risk of infection or relapse. We will be administering a similar ultra-low dose of 2 × 10 ^5^ IU/m ^2^ twice a week.

### Clinical studies of IL-2 in T1D

The first phase I/II study conducted to establish optimal aldesleukin therapeutic dose in adults with T1D was reported in 2013 ^15^. In total, 24 adults with T1D were randomly assigned to aldesleukin or placebo at doses of 0.33 × 10 ^6^, 1.0 × 10 ^6^, or 3.0 × 10 ^6^ IU/day for five consecutive days. A dose-dependent increase in Tregs was detected, with the two lower doses showing more Treg specificity. Treatment with aldesleukin was not associated with any negative effect on glucose metabolism, supporting the safety of using these doses in patients with T1D. Low dose IL-2 upregulated CD25 and FOXP3 on Tregs but not on CD4 ^+^ memory Teffs, and selectively induced pSTAT5 signalling in Tregs ^14^. The proportions of Tregs increased and remained elevated in the trial participants given IL-2 at 1.0 × 10 ^6^ or 3.0 × 10 ^6^ IU at 60 days post-treatment, although levels were lower than those detected soon after the end of IL-2 administration. An injection-site reaction was often seen in treated participants, but no severe adverse effects were reported ^15^.

A completed phase 2b trial in T1D is the ‘Low-dose rhIL-2 in Patients With Recently-diagnosed Type 1 Diabetes (DIABIL-2)’, which is a double-blind randomised placebo-controlled age-stratified (7–35 years old) multicentre European trial assessing efficacy and safety of recombinant human IL-2 in 138 recently-diagnosed T1D patients ( NCT02411253). IL-2 (not aldesleukin but a biosimilar) was administered at a dose of 0.5 MIU/m²/day in children and adolescents in a volume of 1 ml, and 1 MIU/day in adults daily for 5 days and then once every 1–2 weeks between day 15 and 351. (Unpublished data reports that 52 patients have been recruited and treated and the trial end date was 31 March 2019).

In contrast, to characterise dose-response for Tregs of aldesleukin and to find doses that increase Tregs within the physiological range for T1D therapy, our group took a statistical and systematic approach, based on the analysis of the pharmacokinetics and pharmacodynamics of a range of single doses of subcutaneous aldesleukin in the “Adaptive study of IL-2 dose on regulatory T cells in type 1 diabetes” (DILT1D). This was a single centre non-randomised, open label, adaptive dose-finding trial, in 40 adults with a recent diagnosis of T1D ^23^. The primary endpoint was the maximum percentage increase in Tregs from their baseline frequency in each participant measured over the 7 post-treatment days. During an initial learning phase with five pairs, each pair received one of five pre-assigned single doses from 0.04 to 1.5 × 10 ^6^ IU/m ^2^, to model the dose-response curve. Results from each patient were then included into interim statistical modelling in an adaptive design to identify the two doses most likely to induce increases in Treg frequencies of 10% and 20%. The optimal doses of aldesleukin to induce 10% and 20% were 0.101 × 10 ^6^ IU/m ^2^ (95% confidence interval, CI, = -0.0520, 0.254) and 0.497 × 10 ^6^ IU/m ^2^ (95% CI = 0.316, 0.678), respectively.

On analysis of a secondary outcome, the pharmacokinetics of aldesleukin using a recently developed highly sensitive IL-2 assay (5000-fold more sensitive than conventional assays), baseline IL-2 levels were 12.17 – 64.07 fg/ml (0.0007–0.0036 IU/ml), similar to levels in healthy individuals. At 90 minutes post-subcutaneous administration, which is near the time of peak blood concentrations of aldesleukin, IL-2 plasma levels ranged between 0.35 and 27.46 IU/ml (average: 5.73 IU/ml (standard error = 1.07)), depending on the dose delivered. Plasma concentrations of the drug at 90 minutes exceeded the Treg-specific therapeutic window determined *in vitro* (0.015 – 0.24 IU/ml), even at the lowest doses (0.04 – 0.045 × 10 ^6^ IU/m2). We observed a rapid trafficking of Tregs with a dose-dependent decreased frequencies in the circulation at 90 minutes and at day 1, rebounding at day 2 and increasing over the 7-day period to frequencies above baseline. Changes in Teffs, natural killer (NK) CD56 ^bright^ cells and eosinophils were also observed; their frequencies rapidly and dose-dependently decreased in the blood, and then returned to, or exceeded, pre-treatment levels. We also detected a dose-dependent down-modulation of the signalling subunit of the IL-2 receptor, the β chain (CD122), on Tregs and a decrease in their sensitivity to aldesleukin, at 90 minutes and on days 1 and 2 ^23^.

In DILT1D we concluded that the results, most notably a rapid trafficking and desensitisation of Tregs induced by a single aldesleukin injection that resolve within 2–3 days, indicate the following dosing regimen in order to establish a steady-state Treg frequency increase of 20-50%: doses more than 0.1 × 10 ^6^ IU/m ^2^ but not exceeding 0.4 × 10 ^6^ IU/m ^2^, above which Teff expansion is possible, and the interval between dosing greater than every 2 days, and not more than every 7 days. The finding that at 24 hours post-dosing the higher doses of aldesleukin (> 0.38 × 10 ^6^ IU/m ^2^) resulted in sufficient plasma concentrations of aldesleukin to induce Teffs to traffic and proliferate indicated the 0.4 × 10 ^6^ IU/m ^2^ dose as an upper limit in adults to achieve specific Treg increases. Furthermore, giving a dose of this magnitude every day for a few days may activate the effector immune system, and the Treg desensitisation we observed could partly explain the non-responsiveness of Tregs in some adults receiving aldesleukin at a dose of 1.0 × 10 ^6^ IU or more daily ^23^. Hence, we do not support using a daily dosing induction phase of aldesleukin in either adults or children.

The DILT1D trial was followed by the ‘Adaptive study of IL-2 dose frequency on regulatory T cells in type 1 diabetes (DILfrequency)’, to establish the optimal dose and frequency of aldesleukin administration. DILfrequency was a non-randomised, open-label, response-adaptive study of 38 participants with T1D (36 completing treatment), aged 18–70 years, aiming at defining the optimal dose and frequency of IL-2 administration that would lead to sustained increased Treg responses without increasing Teff frequencies ^24^.

The study showed that the optimal regimen to maintain a 30% steady-state increase in Tregs and 25% CD25 expression while avoiding Teff expansion is 0.26 × 10 ^6^ IU/m ^2^ (95% CI -0.007 to 0.485) every 3 days. Tregs and CD25, but not Teffs, were dose-frequency responsive.

Adverse events reported in DILT1D and DILfrequency confirmed the safety of ultra-low dose IL-2. Most participants had transient injection site reactions, consisting of erythema and nodules ^23, 24^.

### Rationale for the ITAD trial

The next step in our customised repurposing of aldesleukin in people with T1D is to test, in children and adolescents with newly diagnosed disease but with remaining endogenous insulin secretion capacity (as measured by circulating C-peptide concentrations), if our dosing regimen can preserve C-peptide levels.

In the proposed phase 2 randomised, double-blind, placebo-controlled clinical trial we will assess whether 6-month treatment with twice weekly doses of aldesleukin, as determined in DILfrequency, can preserve insulin production in children and adolescents diagnosed with T1D within the last 6 weeks. The other unique feature of this trial is the measurement of C-peptide from dried blood spots (DBS) that can be taken at home on a weekly regular basis and stored to be analysed in batches. We have validated this approach against conventional venous blood C-peptide measures and the mixed meal tolerance test (MMTT), both of which are invasive and not feasible in the home setting ^35^. Weekly DBS-based C-peptide testing provides a dense profile of C-peptide changes where the slope of the C-peptide profile over time is the metric on which trial power calculations can be based.

## Protocol

### Objectives

The Primary Objective of the **I**nterleukin-2 **T**herapy of **A**utoimmunity in **D**iabetes (ITAD) trial is to assess the effects of ultra-low dose aldesleukin administration on endogenous β-cell function, as measured by weekly assessments of fasting and post-prandial C-peptide from DBS in children and adolescents with newly diagnosed T1D.

The Secondary Objectives are: 1) To assess the efficacy of regular dosing of aldesleukin in increasing Treg levels; 2) To confirm the clinical safety and tolerability of ultra-low dose aldesleukin; 3) To assess changes in the immune system indicating benefit or potential risk for future gains/loss in β-cell function and immune function; 4) To assess treatment effect on glycaemic control.

Table 1 reports the trial objectives and related outcome measures.

**Table 1.** Study objectives and outcomes.

Objectives	Outcome Measures	Timepoint(s) of evaluation of this outcome measure
**Primary Objective** To assess the effects of ultra-low dose aldesleukin administration on endogenous beta-cell function as measured by frequent home dried blood spot (DBS) fasting and post-prandial C-peptide in children and adolescents with newly-diagnosed type 1 diabetes (T1D).	Differences in slopes of dried blood spot (DBS) C-peptide over the 6 month-treatment period between the active and placebo groups.	Weekly DBS C-peptide collected during the 6-month treatment period, and then monthly during the 6 months of follow-up
**Secondary Objectives** 1) To assess the efficacy of regular dosing of aldesleukin in increasing T-regulatory (Treg) levels	1) Change in Treg, Teff and NK56 ^bright^ cell frequencies from baseline	1) At baseline and then 1, 2, 3, 6 and 12 months from the beginning of treatment
2)To confirm the clinical safety and tolerability of ultra-low dose aldesleukin	2) Safety will be assessed at each visit by: • Physical examination, including assessment of the most commonly reported reactions to low- or high-dose aldesleukin, namely influenza-like syndrome, skin reaction, diarrhoea, nausea; vital signs (temperature, blood pressure, heart rate); weight, abnormal laboratory parameters (liver, kidney function, full blood count); reporting of adverse events.	2) At screening, baseline and then 1, 2, 3, 6 and 12 months from the beginning of treatment
3) To assess changes in the immune system indicating benefit or potential risk for future gains/loss in beta-cell function and immune function.	3) Changes in the absolute numbers of T, B and NK cells. A whole blood 6-color BD TBNK Multitest™ assay using BD Trucount Tubes according to the manufacturers’ instructions (BD Biosciences) will be run to determine the relative and absolute concentration of lymphocyte subpopulations, including T, B and NK cells.	3) At baseline and then, 1, 2, 3, 6 and 12 months from the beginning of treatment
4) To assess treatment effect on glycaemic control	4) Change in HbA1c and daily insulin requirements during the trial period.	4) HbA1c - At baseline and then 3, 6 and 12 months Insulin dose data -Baseline and then 1, 2, 3, 6 and 12 months

**DBS: **dried blood spot; **PBMC:** peripheral blood mononuclear cell; **TBNK FACS:** T-cell, B-cell and NK cell Fluorescence activated cell sorting; **Teff:** T effector cells; **Treg:** T regulatory cells **T1D: **type 1 diabetes

### Study design

ITAD is a phase 2, multicentre, double-blind, randomised, placebo-controlled trial. Children and adolescents (6–18 years) newly diagnosed with T1D (within 6 weeks) will be randomised to receive aldesleukin or placebo, given subcutaneously, twice weekly, 3 or 4 days apart, for a total duration of 6 months. Recruitment to the trial will occur in a minimum of 6 sites across England.

### Intervention

Aldesleukin will be injected subcutaneously at a dose based on body surface area of 0.2 × 10 ^6^ IU/m ^2^ twice-weekly three days apart. Placebo will be injected subcutaneously, at a similar dose (expressed in ml) to the active drug. Where possible, doses should be taken on the same days each week (e.g. Tuesday and Friday or Monday and Thursday). The total amount of time participants will be receiving the aldesleukin/placebo is 6 months.

### Trial participants

The trial population will be represented by 45 children and adolescents, aged 6–18 years, newly diagnosed with T1D. Study participants will be divided in two groups: A (age 12–18 years) and B (age 6–11 years) and recruitment will start with group A. We expect to recruit a minimum of 12 participants in each of the two age groups 12–18 years and 6–11 years. Subjects will be randomised in a 2:1 ratio to aldesleukin or placebo.

After 6 participants from group A have completed a minimum of one month of therapy, there will be a review of safety and efficacy data by the Data and Safety Monitoring Committee and, if no adverse safety signal is observed the recruitment will be widened to include those aged 6–11 years.

Potential participants will be identified from the clinic lists and patient notes at the participating sites. This will be done by the clinical care team.

Infrastructure including several established paediatric clinical sites which are part of the Type 1 Diabetes UK Immunotherapy Consortium and ADDRESS-2 will be used to identify and refer potential participants for ITAD.

Information about ITAD will be posted on the Type 1 Diabetes UK Immunotherapy Consortium and ADDRESS-2 websites along with contact forms so that patients visiting these websites can register an interest in hearing more about the study. The Type 1 Diabetes UK Immunotherapy Consortium/ADDRESS-2 coordination team will refer these patients to the study team at one of the participating research sites for ITAD.

Potential participants will be provided with a verbal explanation of the study and written information sheets. Once they have been given sufficient time to consider their participation in the trial, the participants/parents will be asked to provide written informed consent and their assent to the study.

### Inclusion and exclusion criteria

To be included in the trial participants must: 1) Have given written informed consent to participate or assent with parental consent; 2) Be aged 6–18 years at the time of randomisation (not reached 19th birthday at the time of randomisation; 3) Be diagnosed with T1D (at least one autoantibody positive), requiring insulin treatment; 4) Be within 6 weeks from diagnosis of T1D (at screening); 5) Have a random C-peptide > 200 pmol/l; 6) Have a normal full blood count.

Potential participants may not enter the trial if any of the exclusion criteria listed in Table 2 apply.

**Table 2.** Eligibility criteria.

**Inclusion criteria**
1. Have given written informed consent to participate or assent with parental consent 2. Be aged 6–18 years (not reached 19 ^th^ birthday) at randomisation 3. Be diagnosed with T1D (at least one autoantibody positive), requiring insulin treatment 4. Be within 6 weeks from diagnosis of T1D (at screening) 5. Have a random C-peptide > 200 pmol/l 6. Have a normal full blood count
**Exclusion criteria**
1. Non-type 1 diabetes (type 2 or monogenic diabetes) and secondary diabetes 2. Pre-existing autoimmune disease (excluding type 1 diabetes) 3. Hypersensitivity to aldesleukin or any of the excipients 4. History of severe cardiac disease (NYHA Class III or IV) 5. History of malignancy within the past 5 years (with the exception of adequately treated basal or squamous cell carcinoma or 2. cervical carcinoma in situ) 6. Clinically significant abnormal laboratory values (out of range and associated with clinical symptoms or signs) in haematology, 2. biochemistry, thyroid, liver and kidney function 7. Pre-existing severe major organ dysfunction or seizure disorders 8. Participation in another clinical trial within 4 months prior to screening 9. Females who are pregnant, lactating or intend to get pregnant during the study 10. Females of childbearing potential who are unwilling or unable to comply with contraceptive advice and regular pregnancy testing 2. throughout the trial 11. Sexually active males who are unwilling or unable to comply with contraceptive advice 12. Current use of immunosuppressive agents or steroids 13. Current treatment with hepatotoxic, nephrotoxic, myelotoxic, or cardiotoxic products 14. Active clinical infections – participants can be recruited after a minimum period of 48-h after the last 2. day of feeling unwell or last day of antibiotic/anti-viral treatment 15. Any medical history or clinically relevant abnormality that is deemed by the principal investigator and/or medical monitor to make 2. the participant ineligible for inclusion because of a safety concern 16. Children with compliance problems (families where the local investigators consider that problems with compliance may be an issue)

### Informed consent

The informed consent form has been approved by the Research Ethics Committee and is in compliance with Good Clinical Practice (GCP), local regulatory requirements and legal requirements. The investigator must ensure that each study participant, or his/her legally acceptable representative, is fully informed about the nature and objectives of the study and possible risks associated with their participation.

The investigator will obtain written informed consent from each patient or the patient’s legally acceptable representative before any study-specific activity is performed. Patients under 16 years of age will be provided with an age-appropriate Patient Information Sheet and will be asked to sign an assent form to document their agreement, and their parents will also be asked to provide consent before the child is allowed to participate. Participants aged 16 years and older will be treated as adults and will be able to consent for themselves. Any participants who turn 16 whilst on study, will be re-consented as an adult at their next study visit. The informed consent form used for this study and any change made during the course of this study, must be prospectively approved by the Research Ethics Committee. The investigator will retain the original of each patient signed informed consent form in patient’s medical notes, a copy will be provided to the patient and one copy kept in the trial site file.

Given the relatively large proportion of trial activities that are carried out within the home setting and the number of home visits, it may not be practical to include families who do not have a sufficient level of English comprehension; this is at the discretion of the local investigator. Should a patient require a verbal translation of the study documentation by a locally approved interpreter/translator, it is the responsibility of the individual investigator to use locally approved translators.

### Randomisation and blinding

Consented participants will be randomly assigned in a 2:1 ratio, to receive treatment with the active drug (aldesleukin) or placebo using a centralised randomisation service, Registration/Randomisation and Management of Product (RRAMP), provided by the Oxford Clinical Trials Research Unit. Randomisation will be computer generated using a non-deterministic minimisation algorithm to ensure treatment concealment and balanced allocation across the two treatment groups for site and by age group. The service may be accessed via the secure randomisation website (24 hours/7 days a week).

The patient, local investigator and all study and project management staff will be blinded as to treatment allocation. The statistician will have access to the unblinded code. The site pharmacist will have access to the treatment allocation in order to make up the active drug/placebo.

The RRAMP provides a facility for emergency unblinding of treatment allocation which can be accessed by the local principal investigator. All emergency unbinding will be at the discretion of the local investigators, when clinically indicated for the safety of the patient. Investigators should refer to the standard operating procedures (SOP) for the emergency unblinding procedure provided in the Investigator Site File. Non-emergency unblinding can be requested via the RRAMP system, and will require approval by the Chief Investigator.

## Trial procedures

The study procedures are reported in detail in Table 3. Each participant will be involved in the trial for 13 months, and this includes: Screening phase (1 month), exposure to active drug/placebo (6 months), post-end of treatment follow-up (6 months).

**Table 3.** Schematic representation of assessments at study visits.

	Screening Visit 1	Baseline Visit 2	Visit 3	Visit 4	Visit 5	Visit 6	Visit 7
Day	-1 to-30	0	30	60	90	180	360 Or at study end
Month			1	2	3	6	12
Time window			+/- 7 days	+/- 7 days	+/- 7 days	+/- 7 days	+/- 7 days
**Informed consent**	x						
**Review inclusion/exclusion criteria**	x	x					
**Medical & family history**	x						
**Randomisation**		x					
**Aldesleukin/ Placebo administration**		x ^a^ Twice-weekly, three days apart – with FU call daily for first week					
**Patient AE diary**		Twice-weekly, between treatments. Completed by patient/family at home					
**Provide instructions for DBS at home** **and provision of Ensure Plus**		x					
**Twice-weekly home visits from nurse**		Twice per week to deliver IMP and provide Ensure, if needed.					
**Concomitant medications**	x	x	x	x	x	x	x
**Height, weight and BMI**	x	x	x	x	x	x	x
**Pubertal stage**	x						
**Physical examination**	x	x	x	x	x	x	x
**Vital signs (temperature, Heart rate,** **blood pressure)**	x	x	x	x	x	x	x
**Serum**		x				x ^b^	x
**Random C-peptide (venous)**	x						
**HbA1c (local)**		x ^e^			x	x	x
**HbA1c (central)**		x			x	x ^b^	x
**PBMC**		x	x		x	x ^b^	x
**Treg levels**		x	x	x	x	x ^b^	x
**TBNK FACS assay**		x	x	x	x	x ^b^	x
**DBS for C-peptide ^c^**		weekly at home				Monthly until study end	
**Full blood count ^d^**	x		x	x	x	x	
**Liver function tests ^d^**	x		x	x	x	x	
**Renal function tests ^d^**	x		x	x	x	x	
**Electrolytes ^d^**	x		x	x	x	x	
**Thyroid function ^d^**	x				x	x	
**Pregnancy test (post-menarcheal girls)**	x	x	x	x	x	x	
**Insulin dose data**		x	x	x	x	x	x
**Adverse events review**		x	x	x	x	x	x

AE: adverse event; BMI: body mass index; HbA1c: haemoglobin A1c; IMP: investigational medical product; PBMC: Peripheral blood mononuclear cells; TBNK FACS: T-cell, B-cell and NK cell Fluorescence activated cell sorting; Treg: T regulatory cells ^a^ Treatment should start on day of randomisation or within 7 days thereafter. First dose of IMP or placebo should be given in a hospital setting and the patient observed for a period afterwards. All subsequent doses are administered at home by the research nurse, twice-weekly, three days apart. Wherever possible, doses should be taken on the same days each week e.g. Tuesday and Friday or Monday and Thursday. ^b^should be taken before final injection. ^c^DBS will be also collected at home every week in-between monthly visits until 6 months post treatment start, and then monthly from 7–12 months. Samples should always be taken prior to administration of study drug. The first at-home DBS will be supervised by a research nurse. ^d^Local results may be used if available, and taken within 2 weeks of the visit, or within 4 weeks of the screening/baseline visit. ^e^This should be local HbA1c data, either from point of care (finger prick), or venous samples

## Screening (Visit 1)

Patients need to start screening within 6 weeks of diagnosis of T1D.

**Figure 1.**
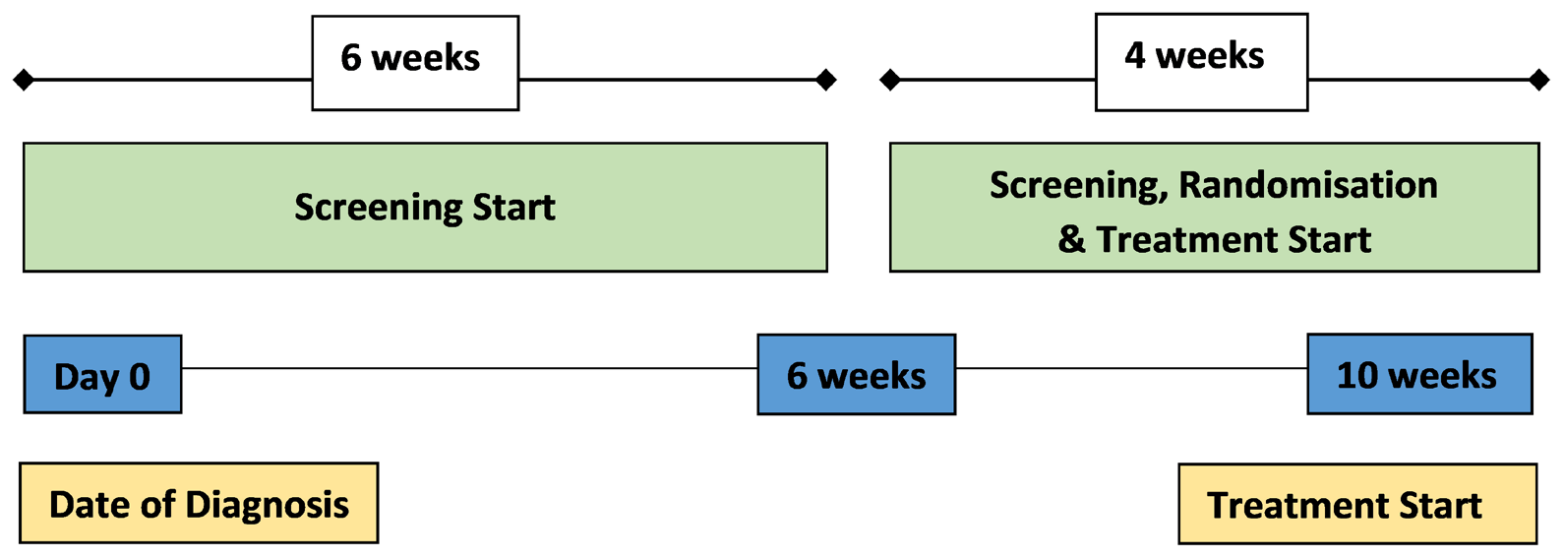
Screening, randomization and treatment start.

Patients will attend an approved participating site for screening within 30 days prior to administration of the first dose of the study medication (Day 1). Screening, randomisation and first dose of trial treatment must be carried out within 10 weeks of diagnosis ( Figure 1).

Assessments performed at screening are as follows: review of inclusion/exclusion criteria, collection of medical history, physical examination (general appearance, cardiovascular system, respiratory system, gastrointestinal system, skin appearance), collection of information on family history and concomitant medications; assessment of height, weight, BMI, pubertal stage, vital signs (temperature, heart rate (HR), blood pressure (BP). Blood samples will be collected for measurement of: C-peptide, full blood count, liver function tests (aspartate transaminase, alanine aminotransferase, total bilirubin, alkaline phosphatase, gamma-glutamyl transferase), renal function tests (creatinine and urea), electrolytes, thyroid function tests (TSH, FT4). A pregnancy test (urine) will also be performed for females of childbearing potential, following menarche.

## Baseline assessments for those who are eligible and have consented (Visit 2)

Following screening, a baseline assessment will be performed and this will include: review of inclusion/exclusion criteria, collection of data on concomitant medications, insulin dose; randomisation; physical examination; assessment of height, weight, BMI, vital signs, blood samples for HbA1c, peripheral blood mononuclear cell (PBMC), TBNK FACS (T-cell, B-cell and NK cell Fluorescence activated cell sorting); DBS for measurement of C-peptide.

At the baseline visit, participants will also be provided with instructions on how to collect DBS for C-peptide weekly at home, including a record of finger prick blood glucose measurements, during the trial period. In addition, they will be provided with: 1) adverse events (AE) diaries and instructions on how to complete them (see extended data ^36^); 2) Ensure Plus liquid meals, or list of standardised breakfasts (see extended data ^36^). Where appropriate, contraceptive advice will be also provided, and a pregnancy test performed.

## Beginning of treatment

Treatment can start on the day of randomisation (visit 2) and should start no later than 7 days after randomisation. Participants will be asked to attend the designated clinical research facility or the hospital site to receive their first injection of aldesleukin or placebo.

Study participants will be observed in the clinical research facility or hospital setting for 2 hours post administration. Participants who experience any adverse event after study drug administration will be directly observed for a further 2 hours or until all symptoms and signs have resolved.

## Subsequent assessments

All subsequent assessments will be performed at the study sites. Assessments will be scheduled to occur ideally on day 3 following the latest injection.

Participants will undergo standard study visits after 1, 2, 3, 6 months from the beginning of treatment (study visits 3 to 6). Each study visit will include: recording of concomitant medications and insulin doses; review of any AE; physical examination; assessment of height, weight, BMI, vital signs (temperature, HR, BP); collection of blood samples for measurements of PBMC, Treg levels, TBNK FACS assay, full blood count, liver function and renal function tests, electrolytes; pregnancy test (where indicated). Additional serum samples will be taken and stored for future analyses. At visit 5 and 6, HbA1c and thyroid function will be also assessed.

For all visits, if there are local blood tests that have been performed as part of standard of care within the two weeks prior to the trial visit, the results of these may be used instead of taking additional samples for trial purposes.

For all visits, if the visit coincides with treatment injection, all blood samples (including DBS sample) should be taken before the injection.

## In between study visits

There will be daily telephone contact with study participants initially for the first week after treatment start and then contact will be via the twice-weekly nurse visits.

In between study visits participants will collect weekly home DBS to measure C-peptide, before and after a liquid meal or standardised breakfast.

A patient AE diary will need to be completed by the participants/their family, twice a week, between treatments, for the duration of their treatment.

### Collection of home dried blood spots

Instruction, both oral and written (provided as extended data ^36^), will be given to participants/parents at the baseline visit about collection of DBS samples at home between hospital visits, and in the first week of treatment, the research nurse will provide further instruction on the DBS collection and the DBS collection will be supervised by them, where possible. Copies of instructions will be provided in the investigator site file.

DBS sampling will be done once a week (+/- 2 days), (before and after a liquid meal or standardised breakfast) from baseline until treatment end at 6 months, and then monthly up to 12 months, for the measurement of C-peptide. If the DBS sample is taken on a treatment day, then the sample must be taken before treatment is given.

DBS samples and blood glucose measurements will be taken fasting immediately before a standardised meal (the first meal of the day), consisting in the Ensure Plus drink, and at 60 minutes from the start of consuming the drink, by finger prick in the home setting. If blood glucose is not between 4 and 11.1 mmol/l, they should take the DBS samples on another day. Participants who are not able to tolerate Ensure Plus will be advised to eat a standardized breakfast with a defined content of carbohydrates, proteins and lipids, which will need to be the same for all DBS collections during the study. Details are provided in the Investigator Site File.

Participants will be asked to withhold their pre-breakfast insulin until after the post-prandial DBS samples have been taken so as not to interfere with the C-peptide result. Following the 60-minute DBS sample, the patient will give a correction dose either via injection or pump, according to the patient’s own insulin sensitivity factor.

The completed DBS card will then need to be air dried for 24 hours before being posted to the laboratory in the pre-paid envelope provided.

DBS collection can be facilitated, when possible, by the research nurses during their twice-weekly home visits to dispense aldesleukin/placebo. Reminders to patients to do the DBS will be provided by the research nurses at their visits. Reminders post-treatment will be given by site staff at participating study sites via a text message or telephone call.

### Adverse events diaries

Participants/parents will be asked to complete AE diaries at home during the treatment period, to monitor AEs associated with the trial treatment.

The diaries should be completed twice a week, between treatments, for the whole 6-month duration of the trial. One questionnaire covers eight injections (approximately one month of treatment).

Paper rulers will be provided in the Investigator Site File that can be given to the families to facilitate measurement of injection site reactions. Completed diaries can be given to research nurses during their visits or can be brought to the next study visit at hospital.

### End of treatment and long-term follow-up assessments

Subjects will be followed up for six months after the end of the 6-month treatment period and during this period they will be asked to collect monthly home DBS to measure C-peptide, before and after a liquid meal or standardised breakfast.

At the end of the 6-month post-treatment follow-up period a formal assessment will be repeated: record of concomitant medications and insulin doses; AE review; physical examination, assessment of height, weight, BMI, vital signs (temperature, HR, BP); collection of blood samples for HbA1c, PBMC, Treg levels, TBNK FACS assay, additional serum samples.

There will be no treatment with aldesleukin after the end of the study. Patients enrolled into this study will already be receiving the appropriate standard of care, and this care will be continued at the end of the study.

### Early discontinuation/withdrawal of participants

During the course of the trial participants or their parents/guardians may choose to withdraw early from the trial treatment at any time. This may happen for a number of reasons, including but not limited to: 1) The occurrence of what the participant perceives as an intolerable AE; 2) Inability to comply with trial procedures; 3) Participant decision. Participants may choose to stop treatment but may remain on study follow-up. Participants may also withdraw their consent, meaning that they wish to withdraw from the study completely. Under these circumstances, the site needs to document all relevant discussions in the patient notes and notify the Trial Office, which will allow the office to mark all future electronic case report forms (CRFs) as not applicable.

Under these conditions, investigators are still responsible to follow up any serious adverse event (SAEs) until resolution. Data collected up to the time of consent withdrawal will be used in the analysis. No further data or samples would be collected after withdrawal.

In addition, the Investigator may discontinue a participant from the trial treatment at any time for any reason including, but not limited to: 1) Pregnancy; 2) Ineligibility (either arising during the trial or retrospectively having been overlooked at screening); 3) Significant protocol deviation; 4) Significant non-compliance with treatment regimen or trial requirements; 5) An AE which requires discontinuation of the trial medication or results in inability to continue to comply with trial procedures; 6) Disease progression which requires discontinuation of the trial medication or results in inability to continue to comply with trial procedures

The type of withdrawal and reason for withdrawal will be recorded in the CRF.

If the participant is withdrawn due to an AE, the Investigator will arrange for follow-up visits or telephone calls until the adverse event has resolved or stabilised.

If a participant is withdrawn from treatment due to pregnancy, the pregnancy will be followed up to outcome.

Any parents or guardian of the participant or the participant requesting to withdraw from intervention will be asked if they want to continue to attend study visits and to complete all study assessments. If they agree then they will be put on to the post-treatment follow-up schedule of monthly DBS and a final visit after 6 months of follow up. During this time all participants will continue to receive the same level of supervision as received by other study patients.

## Intervention

### Legal status of the investigational medical product (IMP)/placebo

Aldesleukin (Clinigen; generic name aldesleukin; trade name, Proleukin) has been available as an approved drug since 1992. The main clinical indication for aldesleukin is treatment of adults (≥18 years of age) with metastatic renal cell carcinoma and metastatic malignant melanoma. In this trial, aldesleukin is being used outside of its licensed indication and being carried out under a Clinical Trial Authorisation. The drug is therefore only to be used by the named Investigators, for the patients specified in this protocol, and within the trial.

The components of the placebo, Water for Injections Ph. Eur., and glucose 5% are both licensed products. Glucose 5% is licensed as a solution for infusion and the water as a solvent for parenteral use. Both are being used within the terms of their licenses.

### Storage and supply of aldesleukin/placebo

The pharmacy at each participating site will prepare and dispense aldesleukin and placebo for this study following instructions received from the Trial Office, and upon receipt of a suitably signed trial-specific prescription.

On receipt of a valid trial prescription containing the subject trial number, date and visit number, the trial pharmacist will first check the treatment allocation through the RRAMP system or refer to the email sent to them upon randomisation to ascertain whether patient is to receive active treatment or placebo.

For each individual participant, a single concentration of the aldesleukin or placebo will be aseptically prepared for administration in individual insulin syringes for each dose.

Each vial of aldesleukin powder for solution for injection contains 22 × 10 ^6^ IU aldesleukin. Aldesleukin contains less than 23 mg sodium per 1 ml and can be considered as 'sodium-free'. The powder is sterile, white and lyophilized. For all injections, aldesleukin is first reconstituted as per aldesleukin summary of product characteristics (SmPC) with water for injection. Solutions are then further diluted to the required concentration in 5% glucose as per the Ceplene SmPC (Noventia Pharma SRL). Vials need to be stored at 2°C to 8°C (in a refrigerator) and protected from light.

Placebo will be prepared with sterile diluent used for the aldesleukin preparation and 5% glucose in appropriately labelled syringes (identical to that used for the diluted active drug) and will be supplied by the study pharmacy to the study nurse/doctor.

Previous data supports stability and sterility of reconstituted diluted IL-2 preparations (reconstituted with as per the aldesleukin SmPC and further diluted with glucose 5% as per the Ceplene SmPC) for up to 14 days at 2–8°C when syringes are prepared by qualified health-care professionals under aseptic conditions and for up to 30 days at room temperature ^37^. However, following Section 10 (Medicines Act), for this study the aseptically prepared product will have an expiry date of 7 days after preparation when stored at 2–8°C. Therefore, syringes containing aldesleukin/placebo will be delivered twice-weekly by the trial research nurses to study participants and the drug either administered by the nurse or self-administered by the participant/carers under the nurse’s supervision.

Syringes will be labelled with trial-specific labelling provided by the trials office that will enable blinding to be maintained. Records of trial treatment allocations will be kept in pharmacy, Drug Dispensing and Accountability Logs will be provided.

All doses that are not used will be returned to the pharmacy at the participating site and a record of their disposal will be made.

### Accountability of the trial treatment

The pharmacies will dispense the drugs to the research nurses (or other members of the study team approved by the Principal investigator), who will deliver the study drugs to the participants. The nurses will be asked by the pharmacists to sign when they collect the IMP and to request the participants’ signature when they deliver the IMP, as well as when patients return the IMP. The nurses will also be asked to sign when they return any unused IMP.

### Compliance with trial treatment

Subject attendance and compliance will be recorded for all visits. At each visit, any unused drug vials will be returned.

### Dose delays and missed doses

Doses should not be delayed, but rather should be skipped. Treatment breaks will be considered based on participants’ individual needs e.g. a planned holiday, by discussion with the Trial Office.

In the circumstances of participants with treatment breaks and those who drop out of the trial an intention-to-treat analysis with secondary sensitivity analyses will be conducted taking these into account, and the sample size accounts for these circumstances.

### Known drug reactions and interaction with other therapies

Most reported AE associated with low dose or ultra-low dose aldesleukin treatment are of mild or moderate severity (grade 1 or 2) ^22^. The main AE reported in patients or healthy volunteers receiving daily doses between 0.18 × 10 ^6^ and 3.0 × 10 ^6^ IU/m ^2^ day have been local reactions at injection sites. At the higher doses, considered as “low dose” versus the “ultra-low” doses for the present trial (0.4 × 10 ^6^ IU/m ^2^ of aldesleukin per week) flu-like syndromes (fatigue, fever, malaise and arthralgia), have been observed (31%) ^22^. Injection site reactions have been reported in around 30–40% of the treated patients, and they seem to be unrelated to the dose and were not seen for all injections in a given patient. Injection site reactions might be related to the propensity of aldesleukin to form aggregates ^22^.

Not clinically relevant abnormalities in biochemical parameters have associated with use of low doses of aldesleukin. Eosinophilia is dependent on the dose and scheme of administration of aldesleukin. It does not occur in all patients and, for the same individual, it does not occur during after all injections ^23^.

In the T1D participants treated in the DILT1D trial, there was an asymptomatic decrease in eosinophils of approximately 15% at 90 minutes followed by an increase on day 1 that resolved by days 3–4. Six participants developed a transient eosinophilia (count >0.4 × 10 ^9^/L) ^23^. Changes in eosinophil count on day 1 was dependent on the additive effects of dose through a model that included both the baseline and dose effects. There was a positive relationship with a higher pre-treatment eosinophil count leading to a greater increase in eosinophils on day 1.

Safety data for ultra-low dose aldesleukin in the paediatric population are available and indicate a low rate of minor AE in this age group ^18^. In a phase 2 paediatric trial, where ultra-low dose aldesleukin (0.1 × 10 ^5^ to 0.2 × 10 ^6^ IU/m ^2^ three times per week) was administered to 12 paediatric patients after allogeneic hematopoietic stem cell transplantation for 6 and up to 12 weeks, AE were all grade 1 only and included muscle aches, arthralgia, fatigue, nausea and decreased appetite. There was no increased risk of infection or relapse.

Some preliminary data from an ongoing paediatric study were recently included in a review on the safety of low-dose IL-2. They indicated that IL-2 was well tolerated in 18 children (7–12 years old) with T1D who were treated with a dose of 0.25 - 1.0 × 10 ^6^ IU per day for a mean duration of more than 6 months ^22^.

More than 13 clinical studies in humans have been completed, including one in healthy volunteers, and others in patients with various conditions: Hepatitis C-induced vasculitis, chronic GvHD, prevention of acute GvHD, T1D, alopecia areata, systemic lupus erythematosus and amyotrophic lateral sclerosis ^13– 30^. Altogether, these studies demonstrated that the increases in Treg number/function were associated with a good tolerance, while an improvement in the clinical outcome was observed in patients with vasculitis, GvHD, alopecia areata and systemic lupus erythematosus. All these studies used Proleukin® (aldesleukin), and the administered doses (by dilution of the formulated Proleukin®) were well below those prescribed for the marketed indications.

The safety of IL-2 is summarized in the aldesleukin SmPC. The doses used in cancer studies are very much higher than those proposed in autoimmune diseases, and the route of administration is most often intravenous. The occurrence of side effects is considerably reduced with the subcutaneous route.

For doses higher than or equal to 10 × 10 ^6^ IU/day administered subcutaneously, SAE are observed; with doses lower than or equal to 3 × 10 ^6^ IU/ day administered subcutaneously no serious adverse reaction has yet been reported.

**Interactions:** Aldesleukin must be avoided in patients taking antineoplastic agents, interferon-alpha therapy, psychotropic drugs ^38^. Medicinal products with known nephrotoxic, hepatotoxic or cardiotoxic potential should be used with caution. Concomitantly administered glucocorticoids may decrease the activity of aldesleukin and therefore should be avoided ^38^. However, patients who develop life-threatening signs or symptoms may be treated with dexamethasone until toxicity resolves to an acceptable level.

Blood pressure should be monitored in patients on treatment with antihypertensive agents, such as beta-blockers, for potential hypotension risk. Contrast media need to be avoided within 2 weeks after treatment with aldesleukin ^38^. In the event of an acute hypersensitivity reaction to aldesleukin, supportive care will be given to the patient according to local clinical protocols.

### Concomitant treatments

All participants will continue to receive intensive insulin treatment, using either multiple daily insulin injection or insulin pump. The patients’ primary physicians will retain responsibility for their diabetes management, and treatment will be prescribed and administered according to local clinical practice at each site, but the research study team can provide close additional support through interaction by phone between study visits as needed.

During the DBS home collection, short or rapid acting insulin should be avoided until the 60- minute sample has been collected.

## Assessment of safety

Safety will be assessed by frequency, incidence and nature of AE and SAE arising during the study.

### Interim safety review

An interim analysis based on safety measures is planned for the age-based step-down approach. The plan is to expose to treatment firstly six subjects in the age group 12–18 years for a minimum of one month. If after review of the unblinded external safety data, satisfactory safety is established, recruitment will be extended to the younger age group, 6–11 years.

### Expected Adverse Reactions (AR)/Serious dverse reactions (SARs)

All expected ARs are listed in the reference safety information, Section 4.8 of the current, approved SmPC ^38^. This must be used when making a determination as to the expectedness of the AR. If the AR meets the criteria for seriousness, this must be reported following the specific requirements.

The relationship of each AE to the trial medication must be determined by a medically qualified individual according to the following definitions:

a) Related: The adverse event follows a reasonable temporal sequence from trial medication administration. It cannot reasonably be attributed to any other cause.

b) Not Related: The AE is probably produced by the patient’s clinical state or by other modes of therapy administered to the patient.

Expectedness will be determined according to the reference safety information, Section 4.8 of the current, approved SmPC.

All AEs occurring during the trial, from randomisation until 30 days after completion of trial treatment, that are observed by the Investigator or reported by the participants, parent/guardian will be recorded on the CRF, whether or not attributed to trial medication.

Clinicians, during the clinic visit AE review, should at a minimum assess: IMP/placebo injection sites (redness, swelling, nodule, and pain), episodes of high temperature, loss of appetite, diarrhoea, nausea, vomiting, reduced activity, chills, headache, and muscle pain.

The following information will be recorded: description, date of onset and end date, severity, assessment of relatedness to trial medication, other suspect drug or device, and action taken. Follow-up information should be provided as necessary.

The local Principal Investigator (or other delegated member of the study team) will review each AE to assess and document the severity of the impact of the event. The severity of events will be assessed on the following scale: 1 = mild, 2 = moderate, 3 = severe.

It will be left to the Investigator’s clinical judgment to decide whether or not an AE is of sufficient severity to require the patient’s removal from treatment. A participant, or his/her parent/guardian, may also voluntarily withdraw from treatment due to what he or she perceives as an intolerable AE. If either of these occurs, the participant must undergo an end of trial assessment and be given appropriate care under medical supervision until symptoms cease, or the condition becomes stable.

### Reporting procedures for serious adverse events

All SAEs will be recorded from IMP/placebo administration and until the final trial visit.

All SAEs must be reported on the SAE reporting form, which will be scanned and emailed to the Trial Office within 24 hours of the Site Study Team becoming aware of the event.

The local Investigator will perform initial assessment of SAE causality on the SAE form. All SAEs will be reviewed by the Nominated Person to determine whether the SAE is related and unexpected. Expectedness will be assessed against the reference safety information, Section 4.8 of the current, approved SmPC. SAEs which may be linked to trial procedures and are unexpected will be recorded as Suspected Unexpected Serious Adverse Reactions (SUSARs).

The Trial Office will perform an initial check of the report, request any additional information, and will pass it on to the nominated clinician without delay. It will also be reviewed at the next Data and Safety Monitoring Committee meeting.

AEs considered related to the trial medication as judged by a medically qualified investigator or the nominated clinician for safety review will be followed either until resolution, or the event is considered stable.

### Events exempt from immediate reporting as serious adverse events

Hospitalisation for elective procedures planned prior to study entry, which have not worsened, do not constitute a SAE.

### Suspected unexpected serious adverse reporting

All SUSARs will be reported by the Chief Investigator to the relevant Competent Authority and to the Research Ethics Committee, and to each participating site, and other parties as applicable. For fatal and life-threatening SUSARs, this will be done no later than 7 calendar days after the Sponsor or delegate is first aware of the reaction. Any additional relevant information will be reported within 8 calendar days of the initial report. All other SUSARs will be reported within 15 calendar days. Treatment codes will be un-blinded for specific patients.

Principal Investigators will be informed of all SUSARs for the relevant IMP for all studies with the same Sponsor, whether or not the event occurred in the current trial.

### Development safety update reports

The Chief Investigator will submit (in addition to the expedited reporting above) Development Safety Update Reports once a year throughout the clinical trial, or on request, to the Competent Authority (Medicines and Healthcare products Regulatory Agency (MHRA) in the UK), Ethics Committee, HRA (where required), Host NHS Trust and Sponsor.

### Pregnancy reporting

Pregnancy testing will be performed, where appropriate, at the beginning and during the trial period up to the last visit while on treatment. All subjects and their parents will be informed of the potential risks and the need for trial patients to use contraception, if sexually active, for the duration of treatment and for 30 days afterwards.

Guidance is in place to deal with any pregnancy, including immediate unblinding and comprehensive independent counselling as to the advisability of continuing the pregnancy.

Participants will be instructed to notify the Investigator immediately if they become pregnant and will be advised to discontinue any study medication immediately. This would be grounds for immediate unblinding to ascertain whether conception had occurred during treatment with placebo or the active drug.

All pregnancies within the trial (either the trial participants or the participant’s partner) should be reported to the Trial Office using the relevant Pregnancy Reporting Form (copies provided in the Investigator Site File) within 24 hours of becoming aware of the event. Pregnancy is not considered an AE unless a negative or consequential outcome is recorded for the mother and/or child/foetus. The outcome of the pregnancy should be recorded and followed up for congenital abnormality or birth defect, at which point it would fall within the definition of “serious”.

## Statistics

### Description of statistical methods

Full details of the statistical analysis will be detailed in a separate statistical analysis plan (SAP) which will be drafted early in the trial and finalized prior to the primary analysis data lock.

Descriptive statistics will be used to describe the demographics between the treatment groups. The primary outcome is change in DBS C-peptide (slopes) during the 6-month treatment period. This will be analysed using Analysis of Covariance (ANCOVA) with adjustment for stratification factors (site, age group) and baseline C-peptide values. Between group differences with corresponding 95% confidence intervals will be reported. If not normally distributed, non-parametric methods will be used with no adjustment (for example the Mann-Whitney Test or Kruskal-Wallis test).

Standard statistical tests will be used to compare the actively treated group with the placebo group (t test, Mann Whitney U-test, analysis of covariance, chi square test), with P values below 0.05 indicating significance.

The incidences of adverse events will be reported and, where appropriate, the two groups will be compared with the use of a logistic regression model.

Analyses will be undertaken on an intent to treat population (all patients analysed as randomised) with sensitivity analyses undertaken to explore deviations from the protocol and compliance with treatment.

An interim analysis based on safety measures is planned for the age-based step-down approach. The plan is to expose to treatment firstly six participants in the age group 12–18 years for a minimum of one month. If after review of the unblinded external safety data and satisfactory safety is established, recruitment will be extended to the younger age group, 6–11 years.

Stata (StataCorp LP) or other appropriate validated statistical software will be used for analysis.

### Missing data

Missing data will be minimised by rigorous data management. Missing data will be described with reasons given where available; the number and percentage of participants in the missing category will be presented by treatment arm. All data collected on data collection forms will be used, since only essential data items will be collected. No data will be considered spurious in the analysis since all data will be checked and cleaned before analysis.

The nature and mechanism for missing variables and outcomes will be investigated, and if appropriate multiple imputation will be used. Sensitivity analyses will be undertaken assessing the underlying missing data assumptions. Any imputation techniques will be fully described in the Statistical Analysis Plan.

### Power calculation

A sample size of 36 patients (24 active drug, 12 placebo) provide 80% power to detect a difference in slopes of DBS C-peptide of 0.53, assuming a two-sample equal-variance t-test, and a between subject standard deviation of 0.523. This SD is based on data on repeated DBS C-peptide from a previous study on 32 children with T1D ^35^.

To account for possible dropouts and for extended periods in which some participants did not receive treatment, the sample size is increased by 25% to 45 children.

During the study, the sample size assumptions will be reviewed by the Data and Safety Monitoring Committee (DSMC) as per the DSMC Charter, who will recommend a change to the sample size if appropriate.

## Data management

The data management aspects of the study are summarized here with details fully described in the Data Management Plan.

### Source data

Source documents are where data are first recorded, and from which participants’ CRF data are obtained. These include, but are not limited to, hospital records (from which medical history and previous and concurrent medication may be summarised into the CRF), clinical and office charts, laboratory and pharmacy records, diaries, microfiches, radiographs, and correspondence.

CRF entries will be considered source data if the CRF is the site of the original recording (e.g. there is no other written or electronic record of data). All documents will be stored safely in confidential conditions. On all trial-specific documents, other than the signed consent, the participants will be referred to by the trial participant number/code, not by name.

To enable peer review, monitoring, audit and/or inspection the Investigator must agree to keep records of all participants (sufficient information to link records e.g., CRFs, hospital records and samples), all original signed informed consent forms and copies of the CRF pages.

### Access to data

Direct access will be granted to authorised representatives from the Sponsor, host institution and the regulatory authorities to permit trial-related monitoring, audits and inspections.

### Data recording and record keeping

All data will be processed according to the Data Protection Act 2018 and General Data Protection Regulation, and all documents will be stored safely in confidential conditions. A data management and sharing plan has been produced for the trial and includes reference to confidentiality, access and security arrangements. All trial-specific documents will refer to the participant with a unique study participant number/code and not by name. Participants’ identifiable data will be stored separately from study data and in accordance with Oxford Clinical Trials Research Unit (OCTRU) SOPs. All trial documentation will be stored securely in offices only accessible by swipe card by the central coordinating team staff in Oxford and authorised personnel.

Data will be collected from participants and local site teams via a secure on-line system called OpenClinica. Participants’ data will be stored and transported in accordance with the Data Protection Act 2018 and OCTRU SOPs.

### eCRF

All data will be entered directly into OpenClinica, which will be accessible online, and data should be entered as soon as possible and in any case no later than one month of the patient visit.

Training on this system can be provided by the Trial Office where needed. All trial data in the CRF must be extracted from and be consistent with the relevant source documents. The CRFs must be completed by the investigator or designee in a timely manner. It remains the responsibility of the investigator for the timing, completeness and accuracy of the CRF pages. The CRF will be accessible to Trial Coordinators, Data Managers, the Investigators, Clinical Trial Monitors, Auditors and Inspectors as required.

The Investigator will also supply the Trial Office with any required, anonymised background information from the medical records as required.

## Samples

### Sample handling

Details of the samples to be collected are listed in Table 2. Details of samples processing, storage and shipment of the samples are provided in the Sample Handling Manual.

The following samples will be analysed and stored at the following laboratories:

a) DBS for C-peptide: Core Biochemical Assay Laboratory (CBAL), Cambridge University Hospitals NHS Foundation Trust, Cambridge; b) Autoantibodies (batch analysis at the end of the trial from serum samples stored at Kings College London): Immunology Laboratory, Cambridge University Hospitals; c) Treg, Teff and NK56 ^bright^ levels/TBNK FACS assay, PMBC isolation, HbA1c (centralized measurement): Autoimmunity and Immunoregulation Laboratory, Kings College London, London; d) HbA1c (local), Full blood count, Electrolytes, Liver and renal function tests, Thyroid function tests, screening C-peptide: Local NHS laboratories.

### Labelling and confidentiality of clinical samples

All samples sent to analytical Laboratories will be pseudonymised/labelled with the study name, study patient number and date taken. Should a laboratory receive any samples carrying unique patient identifiers the recipient must immediately obliterate this information and re-label. The study site will be informed of their error. The trial coordination team will hold all study related information on a secured database that will be only accessed by appropriately qualified, delegated members of the study team.

### Sample retention at end of study

The Chief Investigator has overall responsibility for custodianship of the samples. Laboratories are instructed to retain any surplus samples pending instruction from the Chief Investigator on use, storage or destruction.

It is possible that new or alternative assays may be of future scientific interest. We will ask permission to use any surplus samples, including DNA, in subsequent ethically approved studies in autoimmune disease. Hence, any surplus study samples may be transferred to a licenced tissue bank where they may be stored for an unlimited period of time and will be managed in accordance with applicable host institution policies and the Human Tissue Act requirements.

A patient may withdraw consent at any time. In this event, any samples and data that have already been provided for the research trial will be retained and used in the analysis. No further samples will be taken.

## Quality assurance procedures

The trial will be conducted in accordance with the current approved protocol, Good Clinical Practice (GCP), relevant regulations and SOPs.

This research will be coordinated by the Diabetes and Inflammation Laboratory (DIL) Trial Office and will fall under the UKCRC registered OCTRU, with all personnel working on the trial adhering to OCTRU SOPs. The study may be monitored or audited in accordance with the current approved protocol, GCP, relevant regulations, MHRA and SOPs. A monitoring plan, including risk assessment, has been developed according to OCTRU SOPs. The monitoring activities are based on the outcome of the risk assessment and involve central monitoring and/or on-site monitoring visits.

Regular monitoring will be performed according to GCP. Data will be evaluated for compliance with the protocol and accuracy in relation to source documents. Following written SOPs, the monitors will verify that the clinical trial is conducted, and data are generated, documented and reported in compliance with the protocol, GCP and the applicable regulatory requirements.

All trial staff must hold evidence of appropriate GCP training or undergo GCP training prior to undertaking any responsibilities on this trial. This training should be updated every 2 years or in accordance with Trust policy.

## Monitoring

Regular monitoring will be performed according to the trial specific Monitoring Plan. Data will be evaluated for compliance with the protocol and accuracy in relation to source documents as these are defined in the trial specific Monitoring Plan. Following written standard operating procedures, the monitors will verify that the clinical trial is conducted and data are generated, documented and reported in compliance with the protocol, GCP and the applicable regulatory requirements.

## Serious breaches

The Medicines for Human Use (Clinical Trials) Regulations contain a requirement for the notification of "serious breaches" to the MHRA within seven days of the Sponsor becoming aware of the breach. A serious breach is defined as “A breach of GCP or the trial protocol which is likely to affect to a significant degree: (a) the safety or physical or mental integrity of the subjects of the trial; or (b) the scientific value of the trial”.

In the event that a serious breach is suspected the Sponsor must be contacted within one working day. In collaboration with the Chief Investigator, the serious breach will be reviewed by the Sponsor and, if appropriate, the Sponsor will report it to the Research Ethics Committee, Regulatory authority and the NHS host organisation within seven calendar days.

## Ethics

### Ethical and regulatory guidelines and approvals

The study has received approval from the South Central - Hampshire A Research Ethics Committee, the Health Research Authority and the MHRA and will be carried out following local legal and regulatory requirements. The Ethics Reference is 18/SC/0358. The trial was registered with the European Clinical Trials Database on 6 February 2019 (EudraCT: 2017-002126-20). All substantial amendments to the protocol will be submitted to the appropriate body for approval. The study is sponsored by the University of Oxford.

### Criteria for the termination of the trial

Following any new evidence that arises from previous or current studies, in which, aldesleukin may have serious detrimental effects in the trial population, in the opinion of the Chief Investigator or the Trial Steering Committee, the trial will be discontinued.

### Participant confidentiality

The study will comply with the General Data Protection Regulation and Data Protection Act 2018, which require data to be de-identified as soon as it is practical to do so. The processing of the personal data of participants will be minimised by making use of a unique participant study number only on all study documents and any electronic database(s), with the exception of the CRF, where participant initials may be added. All documents will be stored securely and only accessible by study staff and authorised personnel. The study staff will safeguard the privacy of participants’ personal data.

## Trial committee

The Trial Management Group will be responsible for overseeing the successful conduct and publication of the trial.

A Data and Safety Monitoring Committee will be appointed to safeguard the interests of the trial participants, to assess the safety and efficacy of the interventions during the trial, and to monitor the overall conduct of the trial, protecting its validity and credibility. The Data and Safety Monitoring Committee may advise the Chief Investigator, Trial Steering Committee and Sponsor at any time if, in its view, the trial should be stopped for ethical reasons, including concerns about participant safety or clear evidence of the effectiveness of one of the treatments. The Data and Safety Monitoring Committee will comprise independent medically qualified clinicians, and a statistician.

A Trial Steering Committee will provide supervision of the safe and effective conduct of the trial according to its terms of reference. At least annually, it will review trial progress against agreed milestones, adherence to protocol, participant safety and consider new information. The Trial Steering Committee has the authority to recommend study closure where appropriate.

### Dissemination of information

The trial is registered on Clinicaltrial.gov ( NCT03782636) and EudraCT register ( 2017-002126-20). The trial protocol will be published in an open-access peer-reviewed journal in accordance with the Standard Protocol Items: Recommendations for Interventional Trials statement ( SPIRIT). The trial results will be published in a high-impact open-access journal and presented at relevant international scientific meetings. The trial results will be reported following the Consolidated Standards of Reporting Trials guideline ( CONSORT)

After formal publication, we will inform the participants of the trial results by a specifically designed newsletter. If requested by the participant, their treatment allocation can be revealed once all analyses are complete.

### Trial status

The trial is currently recruiting

## Discussion

Achieving a good glycaemic control represents a main goal in the management of T1D, and insulin therapy remains the cornerstone of treatment ^39^. However, despite continuing improvements in insulin therapy, the majority of children and adolescents with T1D fail to achieve recommended glycaemic targets to reduce risk of short- and long-term complications ^40^.

Preservation of even small amounts of residual endogenous insulin production can improve glucose metabolism, reduce risk of hypoglycaemia and diabetes associated vascular complications, such as retinopathy, nephropathy and neuropathy ^3, 4^. Thus, over the last three decades there have been several immunotherapy clinical trials directed at suppressing the autoimmune response against β cells, rescue residual β-cell mass, and potentially reverse T1D ^41^. However, to date, only a few immunotherapies have shown evidence of slowing C-peptide loss in the short term.

Aldesleukin has been proposed as a good candidate for immunotherapy to prevent or delay autoimmunity based on data from previous genetic studies indicating a major role for the IL-2 pathway in the pathogenesis of T1D ^5, 42, 43^. In addition, IL-2 has a key role in promoting the expansion and function of FOXP3+ Tregs, whose role is critical to prevent autoimmunity ^8^. Tregs respond to lower doses of IL-2 than other cells of the immune systems, such as Teffs or NK cells, because they express higher levels of CD25, and this provides a therapeutic windows for interventions ^8, 12^. It is noted however that NK56bright cells are also highly sensitive to ultra-low dose aldesleukin ^23^; the function of these cells in the context of IL-2 administration in terms of whether they are potentially harmful or beneficial remains to be determined.

Previous studies from our group, namely DILT1D and DILfrequency, have provided valuable information on the optimal dose and frequency of subcutaneous injection of aldesleukin to induce Treg cells expansion and activation without a concomitant deleterious expansion of Teff or NK cells ^23, 24^. These studies, performed in adults with a variable duration of T1D, have also provided reassuring data in terms of short-term safety of ultra-low dose aldesleukin. These positive results provide the rationale and justification for now testing the clinical benefits of our dosing regimen in children and adolescents.

ITAD is the first phase 2 trial using ultra-low dose aldesleukin in children and adolescents with a very recent diagnosis of T1D, who still have detectable C-peptide levels that indicate residual β-cell function, leading to the opportunity of assessing any changes in this C-peptide production because of the exposure to aldesleukin.

Another unique feature of ITAD is the use of a new method of monitoring β-cell function, with frequent C-peptide testing from DBS, which our group recently showed to be a good measure of the status of C-peptide concentrations over time (slope of C-peptide levels) ^35^. Classically, in clinical trials, C-peptide has been measured during a MMTT, which however is labour intensive, requires admission to a clinical research facility and can thus contribute to lower patient recruitment and retention and reduce available blood volumes for secondary analyses ^44^. The DBS method permits frequent C-peptide measurements and monitoring of β-cell function at home. Assessment of changes in the slope of C-peptide through this method is a straightforward and non-invasive approach to assess promising interventions.

The ITAD trial will also provide additional information on the safety of ultra-low dose aldesleukin in the paediatric population over 6 months, which is a longer period of treatment than other studies in T1D, through a strict monitoring of potential AE and assessment of effects on metabolic control and other components of the immune system.

## Data availability

### Underlying data

No data are associated with this article

### Extended data

Figshare: ITAD - additional documents.

This project contains the following extended data: https://doi.org/10.6084/m9.figshare.11932491.v1 ^36^

- Adverse events diaries and instructions.pdf (Study adverse events diary)
- List of standardised breakfasts.pdf (List of standardised breakfast provided to participants)
- DBD Collection.pdf (Dried blood spot collection form and instructions)
- Participant information.pdf (participant information sheets and consent forms)

### Reporting guidelines

Figshare: SPIRIT checklist for ‘Interleukin-2 Therapy of Autoimmunity in Diabetes (ITAD):

a phase 2, multicentre, double-blind, randomized, placebo-controlled trial’. https://doi.org/10.6084/m9.figshare.11950617.v1 ^45^
