# Supplementary material for: Interleukin-2 Therapy of Autoimmunity in Diabetes (ITAD): a phase 2, multicentre, double-blind, randomized, placebo-controlled trial
Source: Wellcome Open Res. 2020 Mar 20;5:49. [Version 1] doi: 10.12688/wellcomeopenres.15697.1 (PMC7194454; doi:10.12688/wellcomeopenres.15697.1)
Supplement: Peer review history

# Peer review: Reviewer response for version 1

**DOI:** 10.21956/wellcomeopenres.17202.r38382

**Published:** 2020-04-21

**Authors:** Steve Bain

**Affiliations:** 1 Diabetes Research Unit Cymru, Swansea University Medical School, Swansea, UK

The study protocol of Marcovecchio *et al*. describes the rationale and design for a phase 2 clinical study in children with recent onset type 1 diabetes. It will examine whether ultra-low dose IL-2 therapy can preserve insulin secretion, in a placebo-controlled trial.

 The regimen chosen, including dose and frequency of administration, has been identified from previous studies which have conducted by the current investigators. These studies compared the effects of various doses, initially tested as a single injection, and then multiple injections in two studies in adults.

 A couple of comments:

 Given that this is a trial which hopes to increase insulin secretion in children with type 1 diabetes, I am surprised that ‘hypoglycaemia’ does not feature more in the collection and reporting of adverse events.

 It is likely that a significant proportion of the children involved in this study will be using continuous glucose monitoring (especially Flash glucose monitoring). It would be a shame to ignore these data and I wonder whether a secondary end-point of time-in-range could be incorporated into the study design?

Is the study design appropriate for the research question?

Yes

Is the rationale for, and objectives of, the study clearly described?

Yes

Are sufficient details of the methods provided to allow replication by others?

Yes

Are the datasets clearly presented in a useable and accessible format?

Not applicable

Reviewer Expertise:

NA

I confirm that I have read this submission and believe that I have an appropriate level of expertise to confirm that it is of an acceptable scientific standard.

# Peer review: Reviewer response for version 1

**DOI:** 10.21956/wellcomeopenres.17202.r38209

**Published:** 2020-04-07

**Authors:** Alberto Pugliese

**Affiliations:** 1 Diabetes Research Institute and Departments of Medicine, Microbiology and Immunology, University of Miami Miller School of Medicine, Miami, Florida, USA

The article by Marcovecchio *et al*. describes the rationale and design for a phase 2 clinical trial in children with recent onset type 1 diabetes. The trial will test whether ultra-low dose IL-2 therapy can preserve insulin secretion, compared to placebo. 

 There is a clear rationale for testing IL-2 in this setting, and in my opinion this therapy has strong potential to become a cornerstone in the treatment of islet autoimmunity, not just for the observed safety, but especially because of the expected benefit associated with improved regulation of the immune system in the absence of conventional immunosuppression. Islet autoimmunity is chronic, and IL-2 regimens can hopefully provide an effective and safe therapy that can be given chronically if needed. 

 The regimen chosen, dose and frequency, has been identified from the previous studies conducted by the investigators. These studies compared the effects of various doses, tested as a single injection first, and then multiple injections to identify a suitable frequency, in two separate studies of adult patients. Certainly, this effort represents a very careful and methodical approach to identify a suitable dose/frequency, and results are being extrapolated to the design of this trial in children.

 There are two innovative aspects to the trial design worth mentioning, the 6 months duration and the use of dried blood spots (DBS) to monitor insulin secretion. Conducting the treatment phase of the trial for 6 months and having the primary outcome at 6 months differs from most trials in this population, which typically have followed patients to their primary outcome for one year. This design offers the obvious advantage of a shorter follow-up period, decreasing cost and patient burden. There is the potential risk that an effect may not become apparent until later, but most of the trials showing benefit in recent onset type 1 diabetes did show an effect by 6 months.

 The use of DBS to monitor fasting and stimulated c-peptide levels (after 60 minutes) is innovative. In an earlier study, the authors had validated this approach against the traditional mixed meal tolerance test (MMTT). There are clear advantages in reducing cost and patient burden, and thus facilitating trial participation and retention. The investigators have likely reasoned about this approach very carefully and many factors must have weighed in their final choice. If cost was not an issue, I would have felt more comfortable if a baseline and 6 months MMTTs were performed as well, to assess the C-peptide area under the curve (AUC). This way, results could have been more directly comparable with the primary outcomes of other trials, and then show equivalence with the DBS results. Also, one must consider that the DBS stimulated DBS samples will be obtained 60 minutes after a standard meal, while the MMTT captures C-peptide data for 2 hours. The primary outcome will be the slope of the change in fasting and stimulated C-peptide from baseline to 12 months. A detail I could not find was whether there are two primary outcomes, fasting and stimulated, or these are combined together, and how would the overall results be interpreted in the event of discordant outcomes in the fasting vs stimulated data. I do like the concept of the frequent, longitudinal assessment of the C-peptide, which would not be possible without using DBS. 

 It is appropriate that investigators plan to continue follow-up to 12 months, and continue to collect specimens for C-peptide and other assessments during the 6 months following the therapy. This would allow to understand whether possible benefits of the therapy continue beyond the treatment window. It was not clear, however, why the assessment of C-peptide at 12 months was not listed as a secondary outcome.

Is the study design appropriate for the research question?

Yes

Is the rationale for, and objectives of, the study clearly described?

Yes

Are sufficient details of the methods provided to allow replication by others?

Yes

Are the datasets clearly presented in a useable and accessible format?

Not applicable

Reviewer Expertise:

Type 1 diabetes, including clinical trials, and have a strong interest in low dose IL-2 therapy.

I confirm that I have read this submission and believe that I have an appropriate level of expertise to confirm that it is of an acceptable scientific standard.
